# Extraction While Swelling of Gums and Toothache

**Published:** 1898-08

**Authors:** 


ARTICLE II.

### EXTRACTION WHILE SWELLING OF GUMS AND TOOTHACHE.

Dr. John M. Parmale presented a symposium of views on the question of the advisability of "Extracting Teeth During Acute Pericementitis." He had sent a circular letter to a number of prominent dentists, asking the following questions :

Assume that a tooth is valueless and condemned to extraction; that it is in a condition of acute periodontitis, an abscess is forming or has formed.

Is it proper at such a time to extract this tooth ?

If yes why ? Are there attending dangers ?

If no, reasons, and course you would pursue.

The query is the result of a talk with physicians, who tell me they often have difficulty in getting dentists to remove teeth at such times.

Dr. Parmale said that about a year ago he had been asked by a physician why dentists would not extract a tooth when it was inflamed. At first he supposed that a tooth was meant that was in a condition that might be treated and the tooth be saved, but found that he meant a tooth that would have to he extracted certainly, and the physician had found that dentists objected to extracting such a tooth at once, but insisted on waiting till the inflammation had gone down.

As a result of that conversation, fifty letters were sent out, and answers received from thirty-four, as to whether it is proper to extract a tooth under the conditions set forth in the query. Affirmative answers were received from all but one of the thirty-four practitioners who responded. The substance of a number of the replies is here given:

Dr. C. E. Francis advised immediate extraction, "to check the trouble as soon as possible, and prevent more serious results, such as increased and more aggravated inflammation, suppuration, and the consequent breaking down or destruction of surrounding tissues." He did not believe there were attending dangers where the operation of removing the teeth is properly performed, and proper precautions are taken to allay any existing inflammation, and the alveolar cavity is rendered free from septic matter.

Dr. J. D. Patterson advised extraction of such a tooth, "because the risks are greater in leaving it in than in extracting; risks of prolonged suffering and the sequele of abscess." He believed there were hardly any attending dangers. His experience taught him that in the extraction of such teeth the inflammatory products, pus, bacteria, etc., are sometimes forced into new territory by the operation, and additional swelling is occasional, but this is temporary, and usually disappears.

Dr. Benjamin Lord stated "that it was his practice to extract, believing that the parts are more likely to be relieved than irritated by the operation; that it is the lesser of two evils."

Dr. G. V. Black advises extraction, and admits the danger of sepsis, but believes this danger is reduced rather than augmented by immediate extraction. He says: "I

am not unmindful of the fact that very grave conditions have developed after extraction in such cases. I have also seen grave conditions follow ordinary extraction, the result of infection. Still the patient is placed in a better condition to ward off danger by the immediate removal of such a tooth, and the possible danger of a graver infection than the one existing at the time should not cause us to hesitate. Of course, proper precautions should be observed, and the after-treatment should be adapted to the conditions."

Dr. Wm. J. Rider says: "I can scarcely understand how any intelligent dentist can refuse to extract a tooth in the condition you speak of. A dead tooth is a foreign member, and inflammation and suppuration is nature's process to throw off anything dead, as tooth, bone, splinter, etc., from the system. By leaving the tooth in, if in the upper jaw, back of the cuspid, there is danger of inflammation of the antrum; if in the lower jaw, of forming a fistula, discharging on the outside. By all means take it out as soon as possible."

Dr. W H. Morgan advocates the extraction of such a tooth, because, by the conditions laid down, no effort is to be made to ultimately save it. He regards the attending dangers as possible, but very remote; and in general he would extract.

Dr. H. B. Noble recommends the extraction of such teeth; has often done it, and never saw or knew of any serious results. In a few cases, where there was considerable swelling, an abscess formed and had to be opened, but he believes that extraction mitigated the pain.—*From Proceedings in Northeastern Dental Association in Dental Cosmos.*

